# Hot Tensile Deformation Behavior and Constitutive Models of GH3230 Superalloy Double-Sheet

**DOI:** 10.3390/ma16020803

**Published:** 2023-01-13

**Authors:** Yiqi Chen, Hong Li, Song Zhang, Jiao Luo, Junfei Teng, Yanlong Lv, Miaoquan Li

**Affiliations:** 1 School of Materials Science and Engineering, Northwestern Polytechnical University, Xi’an 710072, China; 2 Shaanxi Key Laboratory of High-Performance Precision Forming Technology and Equipment, Northwestern Polytechnical University, Xi’an 710072, China; 3 AVIC Manufacturing Technology Institute, Beijing 100024, China

**Keywords:** superalloy sheet, hot tensile deformation, flow behavior, constitutive model

## Abstract

In this paper, the hot tensile deformation of a GH3230 superalloy double-sheet was conducted under deformation temperatures ranging from 1123~1273 K and strain rates ranging from 0.001~0.2 s^−1^. The flow behavior of the GH3230 superalloy double-sheet was analyzed in detail. The hot tensile deformation process of the GH3230 superalloy double-sheet includes four stages of elastic deformation, strain hardening, steady state and fracture. The true stress decreases with the increasing deformation temperature and decreasing strain rate. The variation of the strain rate sensitivity index and strain hardening index with processing parameters were discussed. The average apparent activation energy for hot tensile deformation is 408.53 ± 46.96 kJ·mol^−1^. A combined Johnson-Cook and Hensel-Spittle model considering the couple effect of strain hardening, strain rate hardening and thermal softening was established to describe the hot tensile behavior of the GH3230 alloy double-sheet. Compared to Johnson-Cook model and Hensel-Spittle model, this model has the highest predicting accuracy. The average absolute relative error of true stress between the experimental and the predicted is only 2.35%.

## 1. Introduction

Nickel-based superalloy is an unusual class of metallic materials with an exceptional combination of mechanical properties, high temperature creep, fatigue and resistance to degradation in corrosive or oxidizing environments [1,2]. It has been an essential material for gas turbines in aero engines and power plants [3,4]. GH3230 superalloy is a typical solid solution strengthening alloy with excellent properties. Its microstructure consists of gamma phase, primary granular carbides of M_6_C and granular carbides of M_23_C_6_ at the grain boundary [5]. It is widely used in to manufacture components with double-sheet structure serving in high temperature environments, such as the inner wall of an engine combustion chamber, turbine plate, cooling ring, and so on [6,7].

At present, these components are commonly thin-walled structures, of which the forming methods are hot drawing or hot stamping. The hot tensile formability of the superalloy sheet directly determines the forming quality and service reliability. The hot tensile deformation process of the superalloy sheet is affected by various processing parameters such as deformation temperature, strain rate and strain. The deformation process is complex and is highly nonlinear [8]. An accurate description of flow behavior is important to develop a proper mental-forming process route [9,10]. Up to date, some efforts have been made to understand the tensile deformation behavior of the superalloy sheet [11]. The influence of processing parameters on the flow stress was discussed in detail [12,13]. Some constitutive models have been developed to predict the hot tensile behavior of metal sheets, including the Arrhenius model [14,15,16], the Johnson-Cook (JC) model [17,18,19], the Hensel-Spittel (HS) model [20], the modified JC model [21,22,23], the combined JC and Zerilli-Armstrong model [24], and so on. These models consider different flow behaviors, i.e., strain hardening, dynamic recovery and dynamic flow softening. The prediction accuracies of these models are usually different for different metals.

Hence, in order to get a better understanding of the hot tensile formability of the GH3230 superalloy double-sheet, the flow behavior under hot tensile is systematically investigated. Different constitutive models to describe this behavior are established

## 2. Materials and Methods

The material used in this paper is the GH3230 superalloy double-sheet with a thickness of 2 mm. Its chemical composition (wt%) was 22Cr, 14W, 2Mo, 0.65Mn, 0.35Al, 0.5Si, 0.1C and the balance Ni. The GH3230 superalloy double-sheet was produced by transient liquid phase (TLP) bonding of two cold rolled sheets using a commercial BNi_2_ filler at the bonding temperature of 1323 K. The thickness of these two sheets is 1.2 mm and 0.8 mm, respectively.

**Figure 1:**
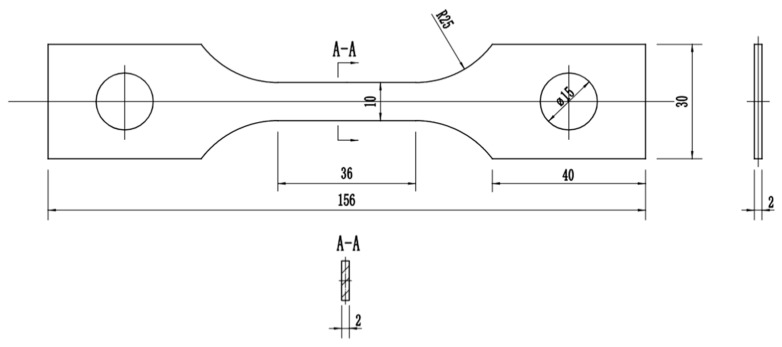
The diagram of hot tensile specimen of GH3230 superalloy double-sheet (mm).

**Figure 2:**
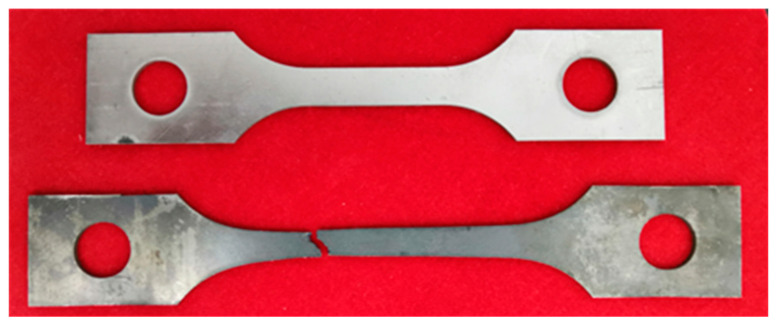
The comparison between original sample and fractured sample of the GH3230 superalloy double-sheet.

The hot tensile experiments of GH3230 superalloy double-sheets were conducted on CMT5105GL electronic universal testing machine based on the China National Standard GB/T 228.2-2015 (Corresponding American standard ASTM E21-20). The specimens for hot tensile with a gauge length of 36 mm and a width of 10 mm were machined along the rolling direction. The diagram of the hot tensile specimen is shown in Figure 1. The comparison between the original sample and the fractured sample deformed after hot tensile deformation of the GH3230 superalloy double-sheet is shown in Figure 2. Prior to loading, the specimen was heated to a given deformation temperature at a maximum heating rate and then held isothermally for 20 min to eliminate the thermal gradient. Subsequently, the specimen was stretched under a given strain rate until it was broken. After tensile testing, it was air-cooled to room temperature. The deformation temperature was selected in the range of 1123~1273 K and the strain rate was selected in the range of 0.001~0.2 s^−1^. During the hot tensile process, the variations of the force and the elongation of the hot tensile specimen were automatically collected and recorded. The extensometer to measure the dimensional change is equipped on the CMT5105GL electronic universal testing machine in which the data gathering from a certain process is controlled by a computer system. Therefore, the relationship between true stress and true strain can be obtained.

## 3. Results and Discussion

### 3.1. The True Stress and True Strain

**Figure 3:**
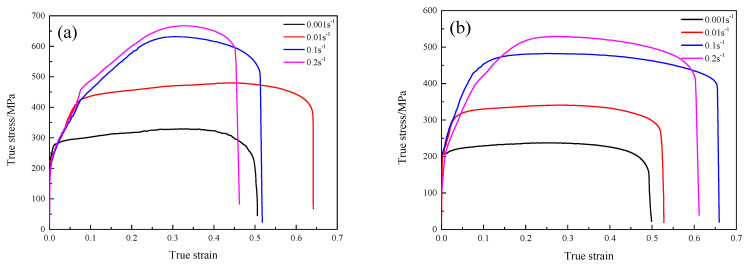

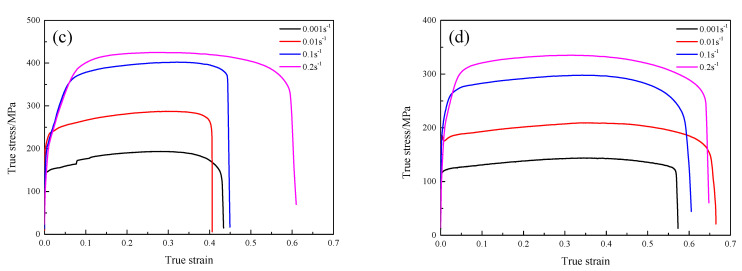
The true stress−true strain curves of the GH3230 superalloy double-sheet during tensile deformation under different temperatures: (**a**) 1123 K; (**b**) 1173 K; (**c**) 1223 K; (**d**) 1273 K.

Figure 3 exhibits typical true stress−true strain curves of the GH3230 superalloy double-sheet during tensile deformation under different deformation temperatures. It can be seen from Figure 3 that the hot tensile deformation process of the GH3230 superalloy double-sheet can be divided into four stages as follows: (1) the elastic deformation stage, in which the true stress increases linearly with the true strain; (2) the strain hardening stage, in which the true stress still increases with the increase of true strain until it reaches a peak value and the increasing velocity is lower than that in the elastic stage; (3) the steady state stage, in which the strain hardening and thermal softening effect are almost balanced and the true stress does not change evidently with the increase in true strain; (4) the fracture stage, in which the true stress decreases rapidly with the increase in true strain until the specimen fractures.

In addition, it can be seen from Figure 3 that the processing parameters significantly affect the flow behavior of the GH3230 superalloy double-sheet during hot tensile deformation. With the increase in deformation temperature, the true stress decreases evidently as the strain rate and the strain are constant. It is because the higher deformation temperature could promote the occurrence of dynamic recrystallization (DRX), which enhances the contribution of thermal softening. With the increase of strain rate, the true stress increases evidently as the deformation temperature and the strain are constant. It is because there is not enough time for thermal softening to offset the contribution of strain hardening induced by dislocation multiplication as the strain rate is higher.

### 3.2. Strain Rate Sensitivity Index

The strain rate sensitivity index is an important parameter to characterize the plastic properties of metal and alloys during plastic deformation. It reflects the ability to resist necking, which is related to chemical composition, microstructure and processing parameters. Generally, a higher strain rate sensitivity index is necessary to obtain good plastic abilities. The strain rate sensitivity index *m* is defined as follows.

(1) $$m=(d\ln\sigma /d\;\ln\dot{\varepsilon })|{}_{\varepsilon ,T}$$

where *σ* is the true stress (MPa), *ε* is the true strain, $\dot{\varepsilon }$ is the strain rate (s^−1^), *T* is the deformation temperature (K). To aid better reading of this article, a notation list is illustrated in Appendix A.

**Figure 4:**
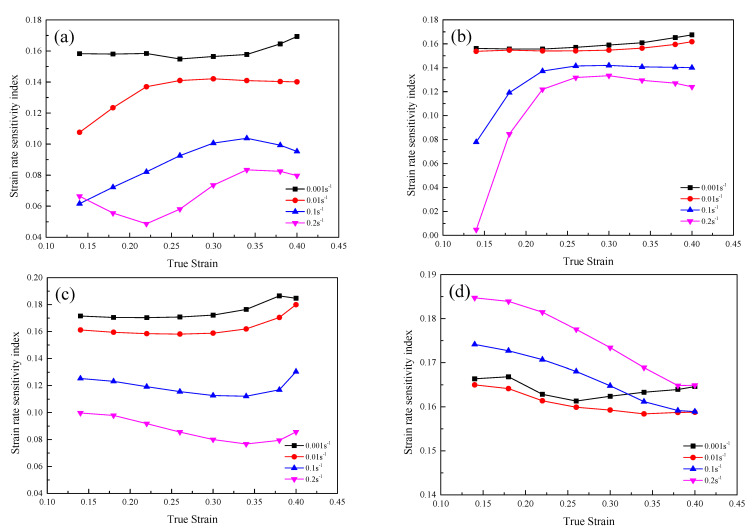
Strain rate sensitivity index of the GH3230 superalloy double-sheet deformed under different deformation temperatures: (**a**) 1123 K; (**b**) 1173 K; (**c**) 1223 K; (**d**) 1273 K.

The effect of true strain on the strain rate sensitivity index of the GH3230 superalloy double-sheet deformed under different deformation temperatures is shown in Figure 4. It can be seen from Figure 4 that the variation of the strain rate sensitivity index of the GH3230 superalloy double-sheet with true strain is determined by the deformation temperature and strain rate. At the strain rate of 0.001 s^−1^ and 0.01 s^−1^, the strain rate sensitivity index almost does not change with the increase of true strain. In addition, the value of strain rate sensitivity index is similar at different deformation temperatures. However, the variation of the strain rate sensitivity index with true strain shows different trends at different deformation temperatures as the strain rate is 0.1 s^−1^ and 0.2 s^−1^. With the increase of true strain, the strain rate sensitivity index increases evidently at a lower deformation temperature (1123 K and 1173 K), while it decreases at a higher deformation temperature (1273 K). In addition, in the deformation temperature range of 1123~1223 K, the strain rate sensitivity index is higher at the strain rate of 0.001 s^−1^ and 0.01 s^−1^ than at the strain rate of 0.1 s^−1^ and 0.2 s^−1^. As the deformation temperature increases to 1273 K, an opposite result is observed. Analyzing the effect of deformation temperature by means of comparing the strain rate sensitivity index ranging of 1123~1273 K, it can be concluded that at the strain of 0.001 s^−1^ and 0.01 s^−1^, the strain rate sensitivity index is less influenced by the increase of the deformation temperature. In the deformation temperature of 1273 K, the strain rate sensitivity index increases significantly, which is ascribed to improved homogeneous deformability caused by the increase in deformation temperature. Therefore, the effect of deformation temperature on the deformability of the GH3230 superalloy double-sheet at a high strain rate is more significant. These results indicate that the GH3230 superalloy double-sheet has a good plastic deformation ability at the deformation temperature of 1273 K. If the deformation temperature decreases, a lower strain rate is required to ensure the plastic deformation.

### 3.3. Strain Hardening Index

The strain hardening index reflects the contribution of deformation resistance via the strain hardening effect. The strain hardening index not only reflects the strain hardening degree of metallic materials, but also reflects the uniform deformation abilities. In the process of hot tensile deformation of the GH3230 superalloy double-sheet, the variation of the strain hardening index is the competition result between the strain hardening effect and the thermal softening effect. The expression of the strain hardening index *n* is shown in Equation (2).

(2) $$n=(d\ln\sigma /d\ln\varepsilon )|{}_{\dot{\varepsilon },T}$$

**Figure 5:**
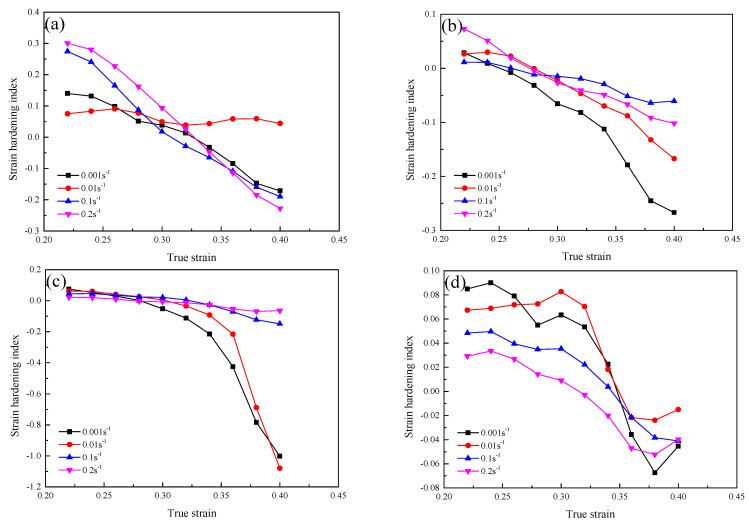
Strain hardening index of the GH3230 superalloy double-sheet deformed under different deformation temperatures: (**a**) 1123 K; (**b**) 1173 K; (**c**) 1223 K; (**d**) 1273 K.

The effect of true strain on the strain hardening index of the GH3230 superalloy double-sheet under different processing parameters is shown in Figure 5. It can be seen from Figure 5 that the strain hardening index decreases evidently as the true strain increases. When the true strain is smaller, the strain hardening index is mostly positive, while it is mostly negative when the strain is higher. This indicates that the contribution of strain hardening is dominant at the starting of hot tensile. As the deformation proceeds, the contribution of thermal softening gradually exceeds the contribution of strain hardening. When the deformation temperature is 1123 K, the increase of true strain has an obvious effect on the strain hardening index at all strain rates. This indicates that the strain hardening is significantly influenced by changes in true strain under a lower deformation temperature (1123 K). When the deformation temperature is 1173 K and 1223 K, the strain hardening index is less influenced by the increase of true strain under a higher strain rate (0.1 s^−1^ and 0.2 s^−1^) because the deformation process is longer when the strain rate is lower. When the deformation temperature is 1123 K, 1173 K and 1223 K, the strain hardening index at high strain rates is larger than that at low strain rates. However, when the deformation temperature is 1273 K, the strain hardening index is the smallest at the strain rate of 0.2 s^−1^. On the one hand, with the increase of true strain, the dislocation density increases continuously, resulting in strain hardening. On the other hand, in the process of hot tensile deformation, because of the cross slip and climbing motion, some dislocations disappear while others are rearranged, which results in the softening. In addition, the strain hardening index is larger when the deformation temperature is lower, which indicates that the strain hardening plays a leading role.

### 3.4. Apparent Activation Energy for Hot Deformation

One of the remarkable characteristics of hot plastic deformation is that the strain rate is controlled by the thermal activation process. The apparent activation energy for hot deformation represents the energy barrier that the atomic transition needs to overcome, which is an important physical quantity to reflect the difficulty of plastic deformation. The apparent activation energy for hot deformation is expressed as follows.

(3) $$\dot{\varepsilon }=A{\left[\sinh\left(\alpha \sigma \right)\right]}^{n}\exp\left(-\frac{Q}{RT}\right)$$

where *Q* is the apparent activation energy for hot deformation (J·mol^−1^), *R* is the gas constant (8.3145 J·mol^−1^·K^−1^), *α* is the material constant (MPa^−1^). The expression of *Q* can be obtained by simplifying Equation (3) as follows.

(4) $${Q=R\frac{\partial \ln\dot{\varepsilon }}{\partial \left\{\ln\left[\sinh\left(\alpha \sigma \right)\right]\right\}}|}_{\varepsilon ,T}{\frac{\partial \left\{\ln\left[\sinh\left(\alpha \sigma \right)\right]\right\}}{\partial \left(1/T\right)}|}_{\dot{\varepsilon }}$$

**Figure 6:**
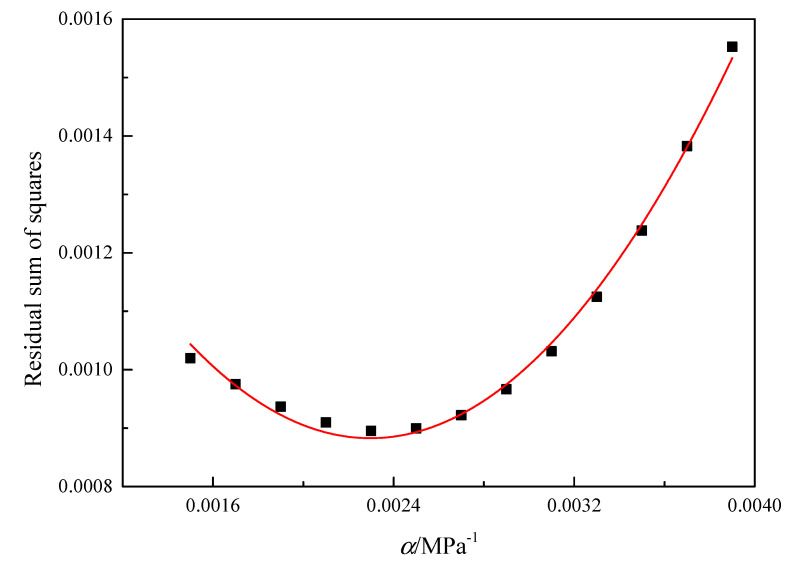
The relationship between the residual sum of squares and α value of GH3230 superalloy double-sheet at the true strain of 0.3.

Based on approximate values of *α*, the values of the residual sum of squares can be calculated by fitting the experimental data through linear statistical regression, and the residual sum of squares should be a parabola function of *α*. Figure 6 shows the relationship between the residual sum of squares and *α* value at the true strain of 0.3, and the optimum *α* value is 0.0023 MPa^−1^. In the same way, the optimum *α* values under different true strains can be obtained.

**Figure 7:**
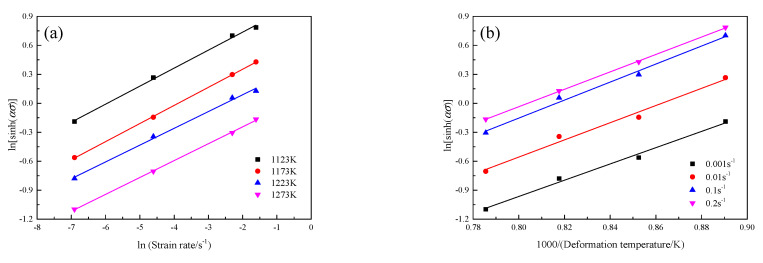
Relationships of (**a**) $\ln\left[\sinh\left(\alpha \sigma \right)\right]-\ln\dot{\varepsilon }$; (**b**) ln[sinh(ασ)]−(1000/T) at the true strain of 0.3.

**Figure 8:**
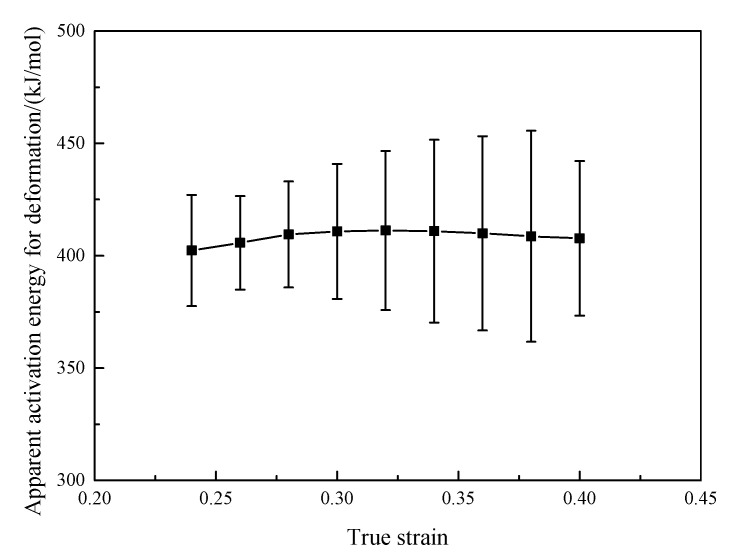
Apparent activation energy for deformation of the GH3230 superalloy double-sheet in hot tensile tests.

Figure 7 displays the relationship of $\ln\left[\sinh\left(\alpha \sigma \right)\right]-\ln\dot{\varepsilon }$ and ln[sinh(ασ)]−(1000/T) at the true strain of 0.3. The value of *Q* can be calculated according to the slope of $\ln\left[\sinh\left(\alpha \sigma \right)\right]-\ln\dot{\varepsilon }$ and ln[sinh(ασ)]−(1000/T). Combing with the calculated *α* value, Equation (4) and Figure 7, the value of *Q* at the true strain of 0.3 is 410.8 kJ·mol^−1^. Figure 8 presents the effect of true strain on the value of *Q* of the GH3230 superalloy double-sheet in hot tensile deformation. As seen from Figure 8, the value of *Q* of the GH3230 superalloy double-sheet slightly varies from 402.3 kJ·mol^−1^ to 411.2 kJ·mol^−1^ with the increasing of true strain from 0.24 to 0.38. It can be regarded as a setting value, which is 408.53 ± 46.96 kJ·mol^−1^.

### 3.5. Constitutive Models

#### 3.5.1. Johnson-Cook (JC) Model

The JC model can be expressed as follows.

(5) $$\sigma =(A+B\varepsilon^{n})(1+C\ln{\dot{\varepsilon }}^{*})(1-T^{*m})$$

where *A* is the yield stress at reference temperature and reference strain rate (MPa), *B* is the coefficient of strain hardening, *C* is the material parameter. ${\dot{\varepsilon }}^{*}=\dot{\varepsilon }/{\dot{\varepsilon }}_{0}$ is the dimensionless strain rate (${\dot{\varepsilon }}_{0}$ is the reference strain rate (s^−1^)), and T^*^ is the homologous temperature and expressed as $T^{*}=\left(T-T_{r}\right)/\left(T_{m}-T_{r}\right)$, where *T* is the deformation temperature (K), *T*_m_ is the melting temperature (K), and *T*_r_ is the reference deformation temperature (*T* ≥ *T*_r_) (K).

In this study, the reference deformation temperature is 298 K and the reference strain rate is 0.001 s^−1^. At the reference deformation temperature and the reference strain rate, the JC model can be simplified to Equation (6).

(6) $$\sigma =A+B\varepsilon^{n}$$

By fitting the true stress−true strain curves of GH3230 superalloy double-sheet at reference deformation temperature and reference strain rate, the parameters *A* and *B* can be obtained. At the reference strain rate, the JC model in the form of Equation (5) can be simplified to the form of Equation (7), and the parameter *m* can be calculated.

(7) $$\sigma =\left(A+B\varepsilon^{n}\right)\left(1-T^{*}{}^{m}\right)$$

**Figure 9:**
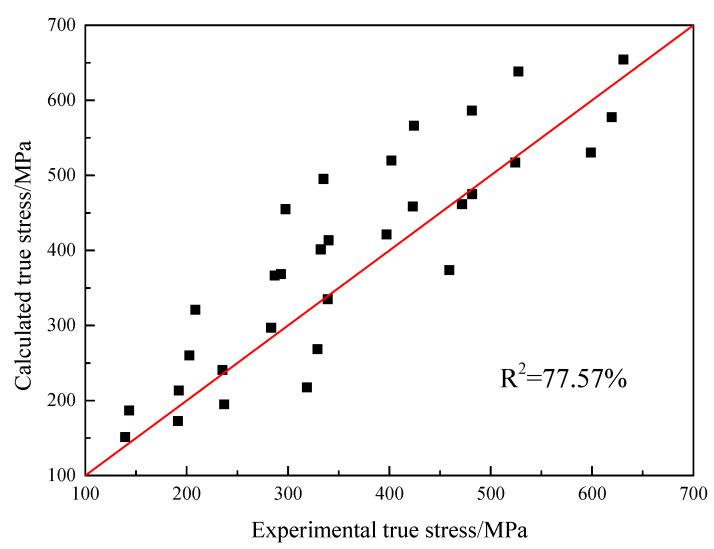
The comparison between the true stress calculated by the JC model and the experimental results.

The parameter *C* can be calculated by combining the data of true strain−true stress in different deformation temperatures and strain rates. The values of the JC model parameters are shown in Table 1. The comparison result between the true stress calculated by the model and the experimental values is shown in Figure 9. The correlation coefficient is 77.57%, which indicates that the present JC model can not to precisely describe the hot tensile behavior of the GH3230 superalloy double-sheet.

**Figure 10:**
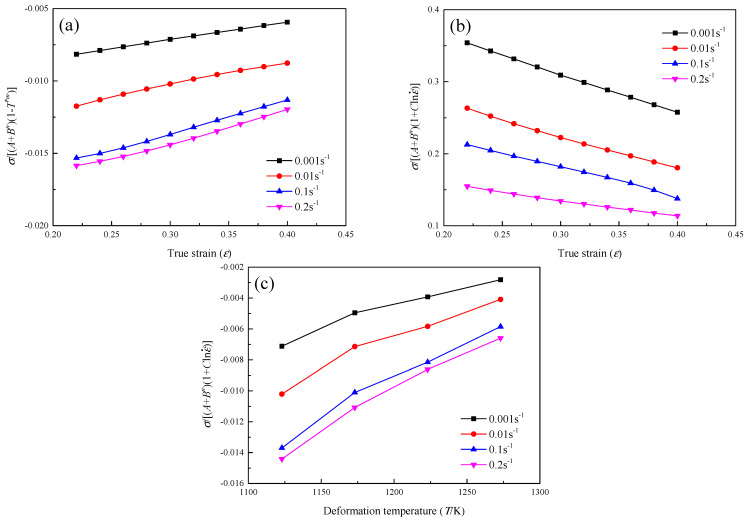
The relationship of: (**a**) $\sigma /\left[\left(A+B\varepsilon^{n}\right)\left(1-T^{*m}\right)\right]-\varepsilon$, (**b**) $\sigma /\left[\left(A+B\varepsilon^{n}\right)\left(1+C\ln\dot{\varepsilon }\right)\right]-\varepsilon$, (**c**) $\sigma /\left[\left(A+B\varepsilon^{n}\right)\left(1+C\ln\dot{\varepsilon }\right)\right]-T$.

In the JC model, the item $\left(A+B\varepsilon^{n}\right)$, $\left(1+C\ln\dot{\varepsilon }\right)$ and $\left(1+T^{*m}\right)$ is used for describing the strain hardening effect, the strain rate effect and the temperature effect, respectively. It is assumed that the strain hardening effect, strain rate hardening effect and thermal softening effect are independent. Based on these assumptions, the calculated $\sigma /\left[\left(A+B\varepsilon^{n}\right)\left(1-T^{*m}\right)\right]$ at the same deformation temperature and $\sigma /\left[\left(A+B\varepsilon^{n}\right)\left(1+C\ln\dot{\varepsilon }\right)\right]$ at the same strain rate will not change with the change of true strain. The calculated $\sigma /\left[\left(A+B\varepsilon^{n}\right)\left(1+C\ln\dot{\varepsilon }\right)\right]$ at the same strain will not change with the change of deformation temperature. Figure 10 shows the relationship of $\sigma /\left[\left(A+B\varepsilon^{n}\right)\left(1-T^{*m}\right)\right]-\varepsilon$, $\sigma /\left[\left(A+B\varepsilon^{n}\right)\left(1+C\ln\dot{\varepsilon }\right)\right]-\varepsilon$, $\sigma /\left[\left(A+B\varepsilon^{n}\right)\left(1+C\ln\dot{\varepsilon }\right)\right]-T$ for hot tensile deformation of the GH3230 superalloy double-sheet. It can be seen that the results predicted by the JC model do not fully reflect the experimental results. The main error of the established JC model describing the hot flow behavior of a GH3230 superalloy double-sheet mainly comes from the lack of consideration of the couple effect between strain hardening, strain rate hardening and thermal softening.

#### 3.5.2. Hensel-Spittel (HS) Model

The HS model with low computational complexity is also used to describe the flow behavior of metals and alloys, which is given as Equation (8).

(8) $$\sigma =A_{HS}T^{m_{1}}\mathrm{Exp}(m_{2}T)\varepsilon^{m_{3}}\mathrm{Exp}(m_{4}/\varepsilon ){\left(1+\varepsilon \right)}^{m_{5}}\mathrm{Exp}(m_{6}\varepsilon ){\dot{\varepsilon }}^{\left(m_{7}+m_{8}T\right)}$$

where *A_HS_*, *m*_1_, *m*_2_, *m*_3_, *m*_4_, *m*_5_, *m*_6_, *m*_7_ and *m*_8_ are material parameters.

Take the natural logarithm of both sides of Equation (8) to get the simplified Equation (9) as follows.

(9) $$\ln\sigma =\ln A_{HS}+m_{1}\ln T+m_{2}T+m_{3}\ln\varepsilon +m_{4}/\varepsilon +m_{5}\ln\left(1+\varepsilon \right)+m_{6}\varepsilon +\left(m_{7}+m_{8}T\right)\ln\dot{\varepsilon }$$

**Figure 11:**
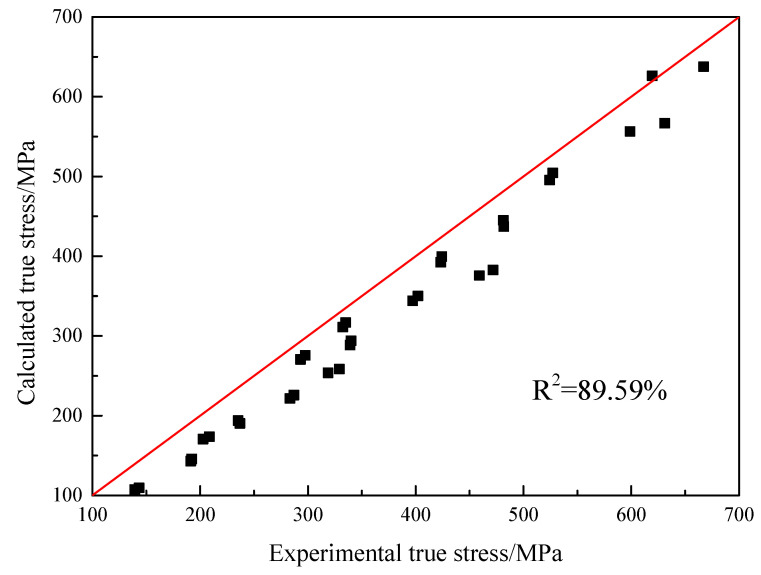
The comparison of calculated values and experimental results of true stress of the HS model.

Based on the true stress−true strain curves of the GH3230 superalloy double-sheet in different deformation temperatures and strain rates, the independent variable matrix is obtained by summarizing the data of true strain, deformation temperature and strain rate. The coefficient matrix is solved by the least square method as follows:

(10) $$\theta ={\left(X^{T}X\right)}^{-1}X^{T}Y$$

where *X* is the independent variable matrix, *X^T^* is the transposed matrix of the independent variable matrix, *X*^−1^ is the inverse matrix of the independent variable matrix, *θ* is the coefficient matrix, *Y* is the dependent variable matrix. Solving the coefficient matrix, the values of the HS model parameters are shown in Table 2. The comparison result between the true stress calculated by the model and the experimental values is shown in Figure 11. The correlation coefficient is 89.59%, which indicates that the prediction accuracy of this model to describe the hot tensile behavior of the GH3230 superalloy double-sheet needs to be further improved.

#### 3.5.3. The Developed Combined JC and HS Model

In this paper, combining the yield and strain hardening portion of the JC model with the temperature, strain hardening and strain rate hardening portion of the HS model, a combined JC and HS (JC-HS) model is developed as given in Equation (11).

(11) $$\sigma =(A+B_{1}\varepsilon +B_{2}\varepsilon^{2})[1+C(\varepsilon )\ln{\dot{\varepsilon }}^{*}]{\mathrm{Exp}\{T}^{*}[m_{1}+m_{2}\ln(1+\varepsilon )+m_{3}\ln\dot{\varepsilon }]\}$$

where *A*, *B*_1_, *B*_2_, *m*_1_, *m*_2_, *m*_3_ are the material parameters, $C\left(\varepsilon \right)=a+b\varepsilon +c\varepsilon^{2}+d\varepsilon^{3}+e\varepsilon^{4}+f\varepsilon^{5}$ is the material parameter associated with true strain, r. ${\dot{\varepsilon }}^{*}=\dot{\varepsilon }/{\dot{\varepsilon }}_{0}$ is the dimensionless strain rate, and T^*^ is the homologous temperature and expressed as $T^{*}=\left(T-T_{r}\right)/\left(T_{m}-T_{r}\right)$. 

**Figure 12:**
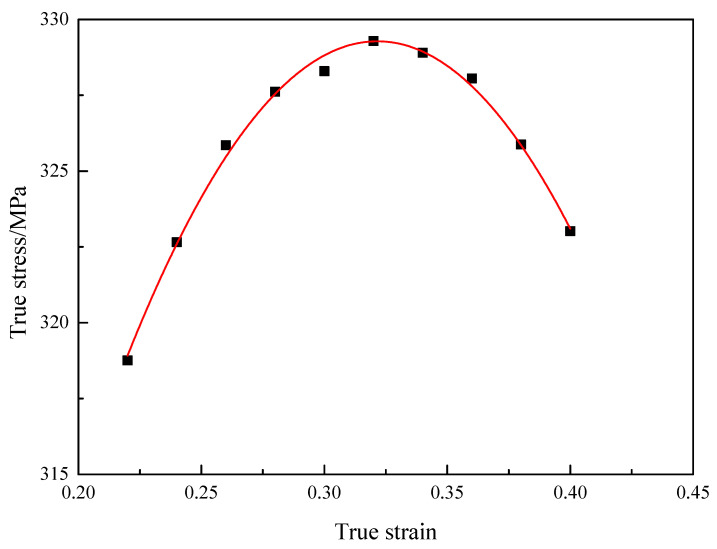
The true stress−true strain curve in reference temperature and strain rate.

For this model, the reference temperature is 1123 K, the melting temperature is 1382 K and the reference strain rate is 0.001 s^−1^. Under the reference deformation temperature and strain rate, the JC-HS model can be simplified to the form of Equation (12), and the true stress−true strain curve in reference temperature and strain rate is shown in Figure 12.

(12) $$\sigma =A+B_{1}\varepsilon +B_{2}\varepsilon^{2}$$

Moreover, Equation (11) can be simplified to the form of Equation (13) under the reference temperature.

(13) $$\left\{ \begin{array}{l} \sigma =(A+B_{1}\varepsilon +B_{2}\varepsilon^{2})[1+C(\varepsilon )\ln{\dot{\varepsilon }}^{*}]\\ C(\varepsilon )=a+b\varepsilon +c\varepsilon^{2}+d\varepsilon^{3}+e\varepsilon^{4}+f\varepsilon^{5} \end{array}\right.$$

**Figure 13:**
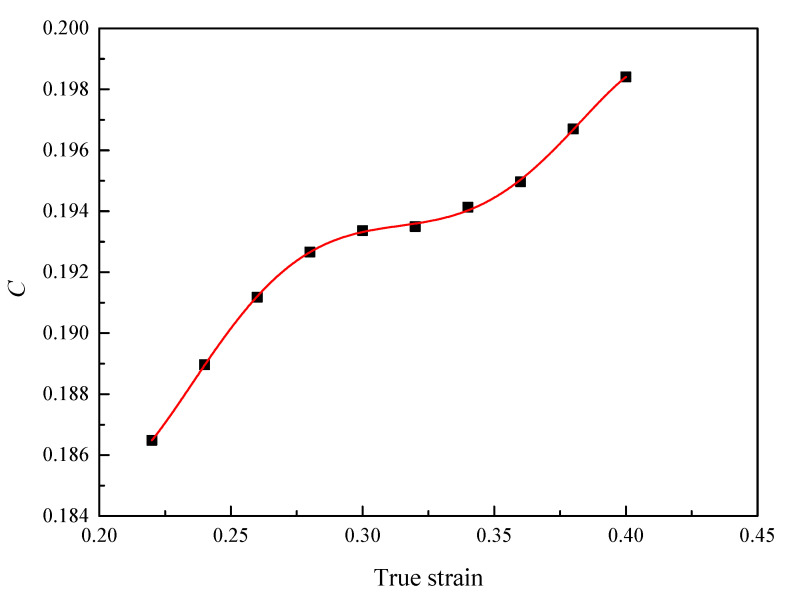
The relationship between *C* and *ε*.

By combining Equations (12) and (13), *C*(*ε*) can be expressed by the slope of the plot of $\sigma =A+B_{1}\varepsilon +B_{2}\varepsilon^{2}$ vs. $\ln\left({\dot{\varepsilon }}^{*}\right)$. The relationship between *C*(*ε*) and strain is shown in Figure 13. The individual parameters in the fifth polynomial of *C(ε)* can be calculated by polynomial fitting, and the values of the parameters are shown in Table 3.

**Figure 14:**
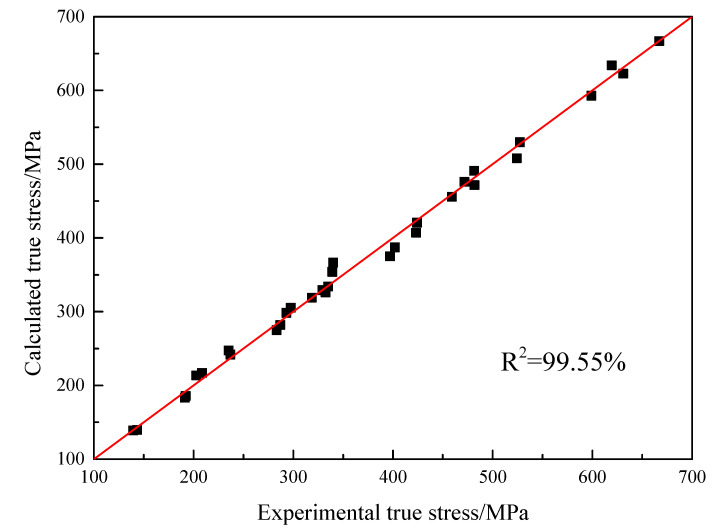
The comparison of calculated values and experimental results of true stress of JC-HS model.

The value of *m*_1_, *m*_2_ and *m*_3_ can be obtained and is respectively equal to −0.005094, −0.002253 and 0.0002098. The comparison result between the true stress calculated by the model and the experimental values is shown in Figure 14. The correlation coefficient is 99.55%.

**Figure 15:**
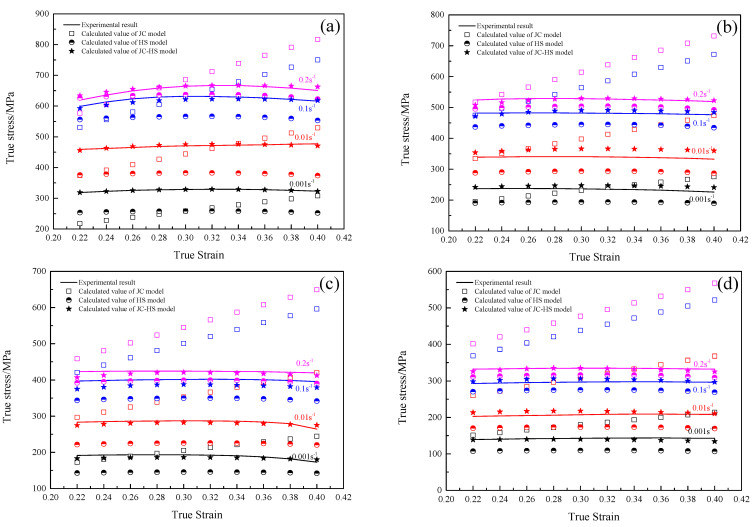
The experimental true stress and calculated by the JC model, HS model and JC-HS model of GH3230 superalloy double-sheet at different deformation temperatures: (**a**) 1123 K; (**b**) 1173 K; (**c**) 1223 K; (**d**) 1273 K.

Figure 15 shows the experimental true stress and the calculated results by the JC model, HS model and JC-HS model for describing the hot tensile deformation behavior of the GH3230 superalloy double-sheet. It can be seen that the predicted true stress by the JC-HS model is closest to the experimental results. The average absolute relative error of true stress between the experimental and that predicted by the JC model, HS model and JC-HS model is 24.60%, 13.51% and 2.35%, respectively.

These results indicate that the prediction accuracy of the JC-HS model is the highest. The established JC-HS model can predict well the hot tensile flow behavior of a GH3230 superalloy double-sheet.

## 4. Conclusions

In this paper, the GH3230 superalloy double-sheet has been tensile deformed in the deformation temperatures ranging from 1123 K to 1273 K, with strain rates ranging from 0.001 s^−1^ to 0.2 s^−1^ until fracture. The hot tensile flow behavior was analyzed and a combined JC-HS constitutive model was proposed. The main conclusions are given as follows.

(1) The hot tensile deformation process of the GH3230 superalloy double-sheet can be divided into four stages, including the elastic deformation stage, the strain hardening stage, the steady state stage and the fracture stage. The true stress decreases with an increasing deformation temperature and decreasing strain rate.
(2) The effect of processing parameters on the strain rate sensitivity index and strain hardening index is significant. The average apparent activation energy for hot tensile deformation of a GH3230 superalloy double-sheet is 408.53 ± 46.96 kJ·mol^−1^.
(3) The combined JC-HS model considering the couple effect of strain hardening, strain rate hardening and thermal softening on the flow behavior of the GH3230 superalloy double-sheet was established. The average absolute relative error of true stress between the experimental and the predicted is only 2.35%. Compared with the JC model and HS model, the JC-HS model has the highest predicting accuracy for describing the hot tensile deformation behavior of the GH3230 superalloy double-sheet.

## Figures and Tables

**Table 1:** Parameters for the JC model for the GH3230 superalloy double-sheet.

*A* (MPa)	*B* (MPa)	*n*	*C*	*m*
354.1	2017	0.878	0.312	0.565

**Table 2:** Parameters for HS model for the GH3230 superalloy double-sheet.

A_HS_/MPa	*m*_1_	*m*_2_	*m*_3_	*m*_4_	*m*_5_	*m*_6_	*m*_7_	*m*_8_
2.7 × 10^−4^	−0.5321	−0.0039	−11.96	−1.000	105.8	−52.57	−0.0541	0.0002

**Table 3:** Reported parameters of the *C*(*ε*).

*a*	*b*	*c*	*d*	*e*	*f*
1.696	−26.62	182.8	−612.4	1004	−646.5

## Data Availability

The data presented in this study are available upon reasonable request from the corresponding author.

## Appendix A

To aid better reading of this article, a notation list is illustrated below.

*σ*	True stress (MPa)
*ε*	True strain
$\dot{\varepsilon }$	Strain rate (s^−1^)
*T*	Deformation temperature (K)
*m*	Strain rate sensitivity index
*n*	Strain hardening index
*Q*	Apparent activation energy (J·mol^−1^)
*R*	Gas constant (8.3145 J·mol^−1^·K^−1^)
*α*	Material constant (MPa^−1^)
*A*	Yield stress (MPa)
*B*	Coefficient of strain hardening
*C*, *A_HS_*, *m*_1_, *m*_2_, *m*_3_, *m*_4_, *m*_5_, *m*_6_, *m*_7_, *m*_8_, *B*_1_, *B*_2_	Material parameter
${\dot{\varepsilon }}^{*}$	Dimensionless strain rate
${\dot{\varepsilon }}_{0}$	Reference strain rate (s^−1^)
*T^*^*	Homologous temperature
*T_m_*	Melting temperature (K)
*T_r_*	Reference deformation temperature (K)
